# p53 Activation by Knockdown Technologies

**DOI:** 10.1371/journal.pgen.0030078

**Published:** 2007-05-25

**Authors:** Mara E Robu, Jon D Larson, Aidas Nasevicius, Soraya Beiraghi, Charles Brenner, Steven A Farber, Stephen C Ekker

**Affiliations:** 1 University of Minnesota, Minneapolis, Minnesota, United States of America; 2 Dartmouth Medical School, Lebanon, New Hampshire, United States of America; 3 Carnegie Institute of Washington, Baltimore, Maryland, United States of America; University of Pennsylvania School of Medicine, United States of America

## Abstract

Morpholino phosphorodiamidate antisense oligonucleotides (MOs) and short interfering RNAs (siRNAs) are commonly used platforms to study gene function by sequence-specific knockdown. Both technologies, however, can elicit undesirable off-target effects. We have used several model genes to study these effects in detail in the zebrafish, Danio rerio. Using the zebrafish embryo as a template, correct and mistargeting effects are readily discernible through direct comparison of MO-injected animals with well-studied mutants. We show here indistinguishable off-targeting effects for both maternal and zygotic mRNAs and for both translational and splice-site targeting MOs. The major off-targeting effect is mediated through p53 activation, as detected through the transferase-mediated dUTP nick end labeling assay, acridine orange, and p21 transcriptional activation assays. Concurrent knockdown of p53 specifically ameliorates the cell death induced by MO off-targeting. Importantly, reversal of p53-dependent cell death by p53 knockdown does not affect specific loss of gene function, such as the cell death caused by loss of function of chordin. Interestingly, quantitative reverse-transcriptase PCR, microarrays and whole-mount in situ hybridization assays show that MO off-targeting effects are accompanied by diagnostic transcription of an N-terminal truncated p53 isoform that uses a recently recognized internal p53 promoter. We show here that MO off-targeting results in induction of a p53-dependent cell death pathway. p53 activation has also recently been shown to be an unspecified off-target effect of siRNAs. Both commonly used knockdown technologies can thus induce secondary but sequence-specific p53 activation. p53 inhibition could potentially be applicable to other systems to suppress off-target effects caused by other knockdown technologies.

## Author Summary

Recent advances in sequence-based approaches to “knockdown” gene function have opened the door to an array of approaches to uncover functions for genes of interest. Vertebrate knockdown strategies—such as morpholinos (MOs) in zebrafish or RNA interference-based strategies in mammalian systems—have been demonstrated to be effective, rapid, and cost-efficient reverse-genetic approaches for studying gene function. However, their deployment has to date been limited by a number of technical (genomic, biological, and off-targeting) hurdles. One of the notable and unexpected findings from our work using MOs has been a series of observations surrounding unanticipated effects that are independent of the intended gene target. We have identified and characterized a recently described p53 induction pathway due to off-targeting that appears to be shared between knockdown technologies. This study reconciles a series of unexpected findings that show p53 upregulation at the transcriptional level in a subset of short inhibitory RNA- and MO-treated vertebrate systems. Moreover, concurrent p53 knockdown provides a new approach to facilitate the identification of previously hidden gene functions. This study provides both a new gene knockdown enhancement tool as well as additional insight into an important and conserved pathway implicated in cellular toxicity.

## Introduction

Morpholino phosphorodiamidate oligonucleotides (MOs) [1] and short inhibitory RNAs (siRNAs) [2] have been instrumental to induce sequence-specific gene knockdown in multiple systems. However, the use of both technologies is sometimes limited by induction of off-target effects [3–7]. About 15–20% of MOs used in zebrafish show off-targeting effects [3], represented by a signature neural death peaking at the end of segmentation (1 day post-fertilization [dpf]). The affected embryos grow with smaller heads and eyes, exhibit somite and notochord abnormalities, and eventually display craniofacial defects. These MO-induced developmental defects are target-independent because they are not displayed by characterized mutants in the respective genes [3].

We show here that the off-target effects of MOs are mediated through p53-induced apoptosis. Concurrent knockdown of p53 with various MOs significantly alleviates off-target neural death. Importantly, however, p53 MO did not affect specific phenotypes induced by a variety of MOs. We propose the use of p53 knockdown as a tool to attenuate off-target effects and facilitate the study of specific loss of function phenotypes.

## Results

### MOs Exhibit Off-Target Neural Apoptotic Effects

**Figure 1:**
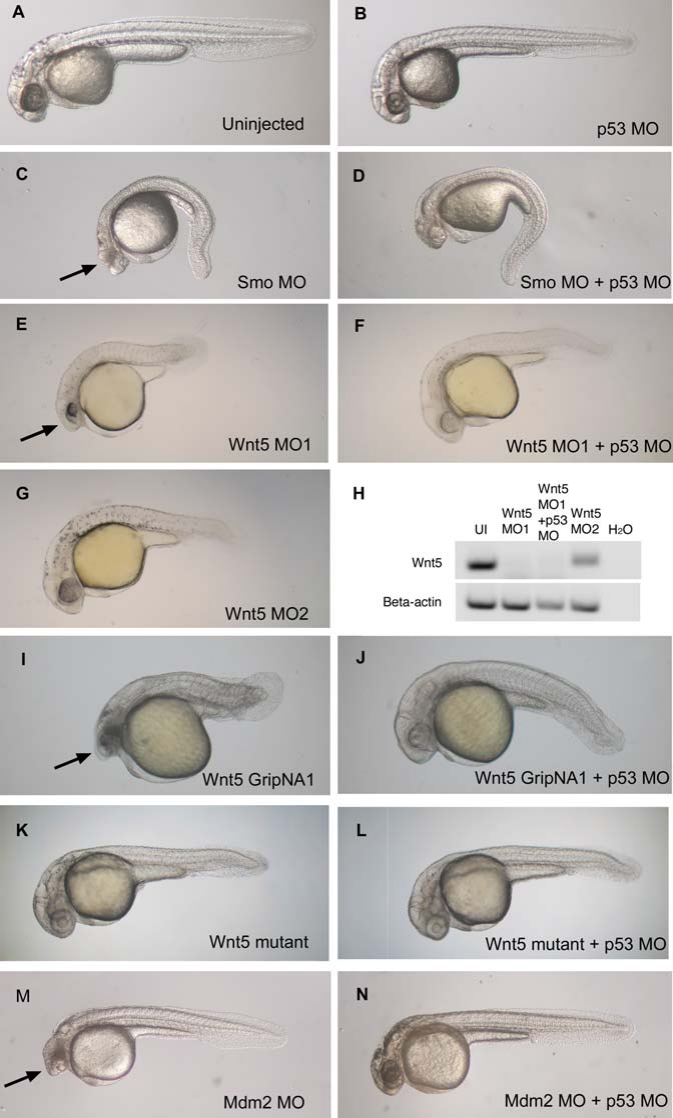
p53 MO Attenuates Cell Death Induced by MOs and GripNAs (A–G and I–N) Brightfield images of 28 hpf embryos injected with demonstrative MOs and gripNAs. The arrows (C, E, I, and M) indicate neural death that is significantly attenuated to normal head size and morphology by co-knockdown of p53 (D, F, J, and N). Interestingly, Wnt5 MO2 shows no significant neural death (G) even at 6 ng, a higher dose than Wnt5 MO1 (E) (3 ng), but can elicit a highly penetrant Wnt5 phenotype even at 1.5 ng (unpublished data). (H) Effect of p53 MO on Wnt5 splicing. We carried out RT-PCR using primers spanning the exon 5–exon 6 junction targeted by Wnt5 MO1. Proper splicing was completely inhibited by Wnt5 MO1. p53 co-knockdown did not affect the efficiency of Wnt5 MO1 inhibition. Embryos injected with Wnt5 MO2, which targets the previous junction (exon 4–exon 5), still exhibited some properly spliced transcript at the exon 5–exon 6 junction. β-actin was used as a loading control.

General morphological features of MO-induced off-target neural death have been previously described [3]. We further investigated the nature of this cell death and the mechanism of MO mistargeting. For this report, we focused primarily on MOs designed against two gene targets for which mutants have been previously described, *wnt5*/*pipetail (ppt)* [8] and *smoothened*/*slow muscle omitted (smu)* [9,10], to facilitate the discrimination between specific and nonspecific effects. A translational MO against *smoothened* (Smo MO) induces characteristic *smu* phenotype (spinal curvature, U-shaped somites) (Figure 1C). A splice-site *wnt5 MO* (Wnt5 MO1) induces tail and body-axis shortening and somite compression (Figure 1E), characteristic of the *wnt5/ppt* mutant (Figure 1K). What both Smo MO- and Wnt5 MO-injected embryos (morphants) have in common is an additional and very similar neural death (Figure 1, arrows). This neural death is target-independent, since it is not exhibited by the respective mutants (Figure 1K) [9,10]. Nonetheless, this neural death appears to be sequence-specific, since a completely different splice-site *wnt5* MO (Wnt5 MO2) shows no neural death, but readily induces the characteristic *wnt5/ppt* phenotype (Figure 1G). We tested another type of knockdown molecule based on an alternating trans-4-hydroxy-L-proline/phosphate polyamide backbone called gripNA [11]. Interestingly, a gripNA targeting *wnt5* (similar in sequence to Wnt5 MO1) also induces neural death along with the characteristic *wnt5/ppt* phenotype (Figure 1I). A gripNA against *smoothened* also causes additional neural death (unpublished data), supporting the idea that the off-targeting effects are not limited to the MO chemistry, but represent a common feature to these knockdown technologies.

**Figure 2:**
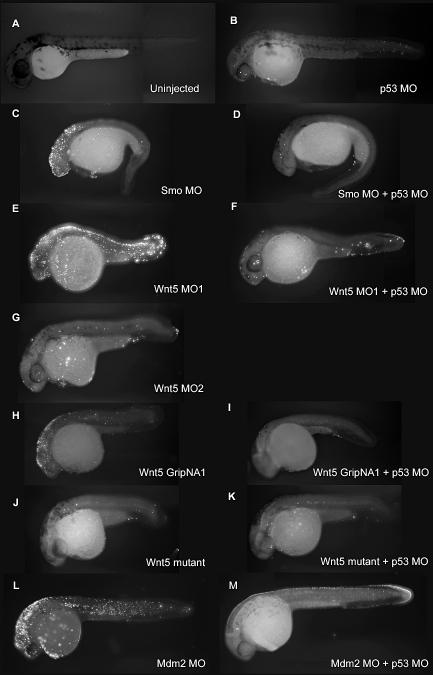
p53 MO Attenuates Apoptosis Induced by MOs and GripNAs, as Detected by TUNEL Assay (A–M) Fluorescent images of 30 hpf embryos injected with indicated MOs and subjected to TUNEL assay to detect apoptosis. A strong fluorescent signal for Smo MO (C), Wnt5 MO1 (E), and Wnt5 GripNA1 (H), similar to Mdm2 MO (L), indicates increased apoptosis. The fluorescent signal is strongly diminished by co-knockdown of p53 (D, F, I, and M), similar to the uninjected control (A), Wnt5 MO2 (G), or Wnt5 mutant (J). Wnt5 mutant is not affected by p53 knockdown (K).

**Figure 3:**
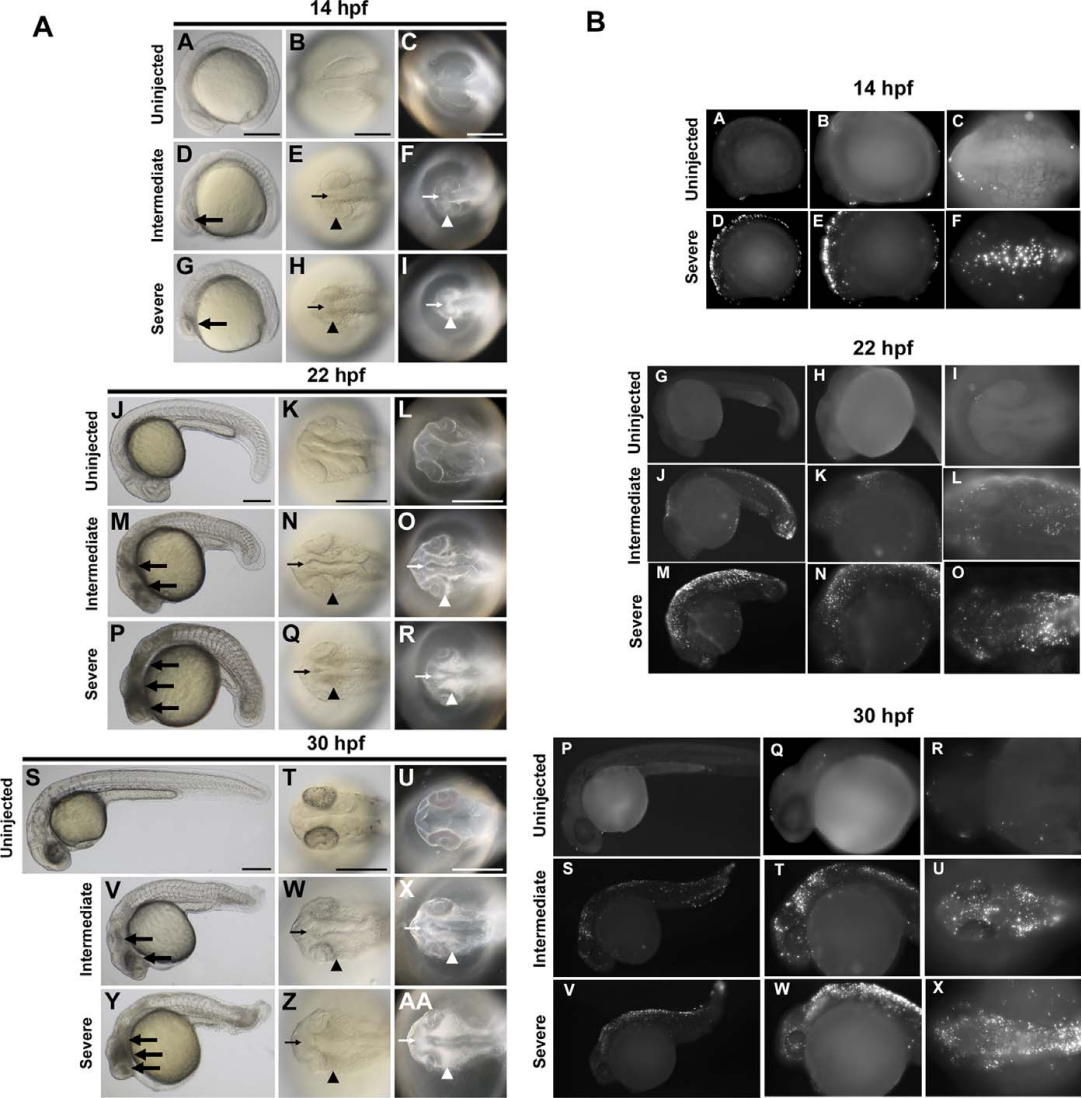
Temporal and Spatial Characterization of Representative MO-Induced Neural Cell Death during Early Embryogenesis (A) Brightfield and darkfield images of Wnt5 MO1-injected embryos. 14 hpf (A–I), 22 hpf (J–R), and 30 hpf (S–AA). Uninjected embryos (A–C, J–L, and S–U), intermediate cell death phenotype (D–F, M–O, and V–X), and severe cell death phenotype (G–I, P–R, and Y–AA). Lateral views (A, D, G, J, M, P, S, V, and Y), all others dorsal head views. Intermediate cell death is observed at 14 hpf as highly localized opaque cells in the head (large arrow in D), which are arranged near the lateral (arrowhead in E and F) and midline (small arrow in E and F) areas of the developing brain. This pattern progresses through 22 hpf and 30 hpf (M–O and V–X, respectively), including a concentration of opaque cells surrounding the emerging folds of the brain midline (small arrows N–O and W–X) and the eye (arrowheads N–O and W–X). Severe cell death is observed as highly dense areas of opaque cells throughout the developing head. (B) TUNEL assay. Zebrafish embryos were injected with Wnt5 MO1 and analyzed by TUNEL assay at 14 hpf (A–F), 22 hpf (G–O), and 30 hpf (P–X) stages. Uninjected embryos: A–C, G–I, and P–R. At the later time points two classes of phenotypes were observed: an intermediate (J–L and S-U) and a severely affected class of embryos (M–O and V–X). These were characterized by intense fluorescent apoptotic foci in the head and body, with increasing intensity corresponding to increased severity (higher MO dose). Please see Figure S1 for a higher resolution version of this figure.

**Figure 4:**
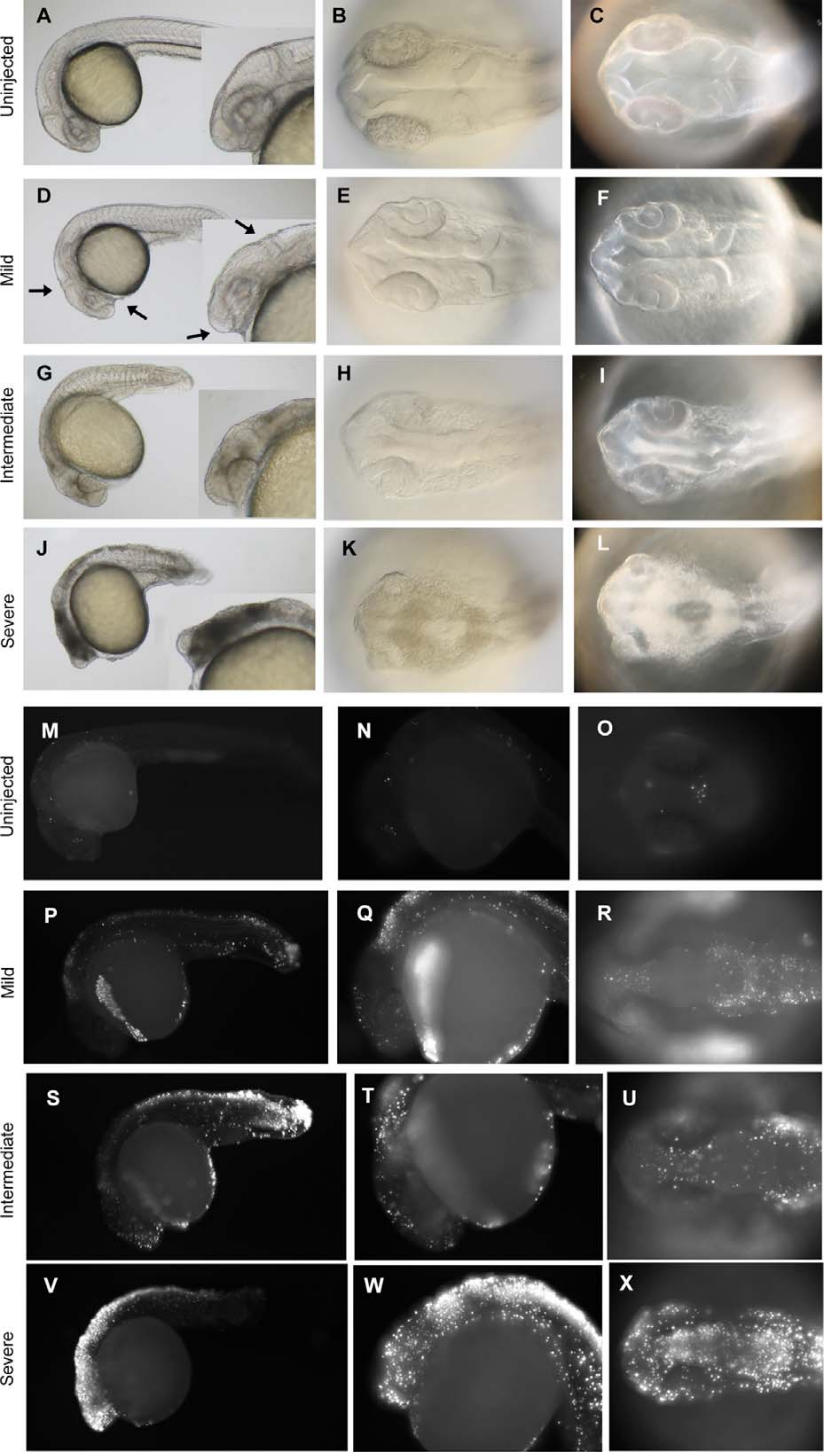
Representative Cell Death Phenotypes Detected at 26 hpf Brightfield (A, B, D, E, G, H, J, and K) and darkfield images (C, F, I, and L) of 26 hpf zebrafish embryos injected with Wnt5 MO1. (M–X) TUNEL assay. Lateral views (A, D, G, J, M, N, P, Q, S, T, V, and W), with inserts showing a higher magnification of the head region (A, D, G, and J). Dorsal view of the head (B, C, E, F, H, I, K, L, O, R, U, and X). The two classes of phenotypes described in Figure 3 are observed at this time point, too (intermediate, G–I and P–R; severe, J–L and S–T; uninjected embryos, A–C and M–O). At 26 hpf, however, a milder cell death phenotype was also observed (D–F). This class of embryos exhibited an anterior-ventral concavity and a depressed hindbrain (indicated by the arrows), without tissue with obvious characteristic cell-death patterns featured by embryos with intermediate and severe phenotypes (compare [F] to [I] and [L]). However, even these mildly affected embryos showed clear apoptosis indicated by the TUNEL assay (P–R).

The off-target neural death induced by MOs is highly reminiscent of the neural death induced by a published Mdm2 MO (Figure 1M). Mdm2 is a negative regulator of the tumor suppressor p53, the gene most frequently mutated in human cancers [12]. Mdm2 knockout in mice is an embryonic lethal [13] due to extensive p53 upregulation and p53-induced apoptosis. Mdm2-targeted MO in zebrafish was reported to induce apoptotic neural death [14]. We examined the mechanism of MO-induced off-target neural death by testing for apoptosis in multiple MO-injected zebrafish embryos using a transferase-mediated dUTP nick end labeling (TUNEL) assay (Figure 2) and by staining with acridine orange (unpublished data). Our results suggest that the neural death induced by off-targeting MOs is apoptotic in nature and was indistinguishable from the cell death observed in the Mmd2 knockdown [14]. We tested the specificity of Mdm2 MO-induced cell death by overexpressing a Mdm2 RNA construct. However, we did not observe any significant rescue of the Mdm2 MO-induced cell death with the Mdm2 RNA construct (unpublished data). Therefore, it is possible that the cell death phenotype induced by the Mdm2 MO is also primarily an off-targeting effect. We performed an in-depth analysis of MO off-targeting to examine the concordance between the phenotypes observed by light microscopy and apoptosis patterns observed by TUNEL staining. We analyzed zebrafish embryos injected with Wnt5 MO1 at 14 hpf (the onset of cell death, [3]), 22 hpf, 26 hpf, and 30 hpf (Figures 3 and 4; Figure S1). Brightfield images show the signature appearance of opaque-looking discolored tissue around the eyes and in the nervous system in embryos injected with Wnt5 MO1 (Figures 3 and 4; Figure S1). The extent of the opaque tissue increased at later time points and with increasing MO dose. The cell death could be more easily visualized using darkfield microscopy (Figures 3A, 3C, 3F, 3I, 3L, 3O, 3R, 3U, 3X, 4C, 4F, 4I, 4L, 4O, 4R, 4U, and 4X). This analysis shows the characteristic pattern of white tissue corresponding to the opaque structures seen in brightfield that is diagnostic of MO mistargeting in zebrafish embryos.

At 26 hpf, milder phenotypes displaying a characteristic anterior-ventral concavity and/or hindbrain depression could be observed, in the absence of the opaque/white tissue characteristic of the more severe cases of cell death (Figure 4D–4F). However, when analyzed by TUNEL staining, we observed that even the mild phenotypes (as seen by light microscopy) were associated with significant apoptosis (Figure 4P–4R). These mildly affected embryos usually recovered by 30 hpf, when they showed significantly less cell death, if any (unpublished data). The more severely affected embryos did not recover until day 2 or 3, at which time the characteristic apoptotic tissue was no longer apparent either through light microscopy or TUNEL analysis (unpublished data). However, these embryos lacked some neural tissue and developed with smaller heads and eyes (unpublished data).

The punctuated pattern of neural apoptosis seen at all time points and with increasing intensity in the more severe cases was strikingly different from the normal apoptotic pattern seen in uninjected control embryos (Figures 3 and 4). Developmentally regulated apoptosis has been described in detail [15] and was recapitulated by our analysis (Figures 3 and 4, uninjected embryos). However, at all studied time points the extent of developmentally regulated apoptosis was significantly less extensive than the apoptosis induced by MO off-targeting. In particular, at 30 hpf, little if any apoptosis was noted in control embryos. Therefore, we performed the TUNEL analysis at 30 hpf for all subsequent experiments to clearly differentiate between developmentally regulated apoptosis and apoptosis caused by MO off-targeting.

### p53 MO Attenuates Off-Target Neural Death Induced by MOs

The neural apoptosis induced by a variety of MOs and the similarity to the phenotype induced by apparent p53 upregulation (Mdm2 MO) suggested the hypothesis that MO off-target effects can induce the p53 apoptosis pathway. Therefore, we tested whether p53 knockdown can rescue the off-target apoptosis phenotype induced by several MOs. Indeed, p53 MO attenuated the neural death induced by smoothened and Wnt5 MOs, as shown by morphology (Figure 1D and 1F), acridine orange (unpublished data), and the more specific TUNEL assay (Figure 2D and 2F). Similar results were observed for p53 knockdown rescue of Mdm2 MO-induced apoptosis (Figures 1N and 2M). Interestingly, p53 MO also alleviated the neural death induced by the Wnt5 gripNA, suggesting that this additional knockdown technology can upregulate the p53 pathway due to off-targeting (Figures 1J and 2I). However, as expected, p53 MO did not have any effect on *wnt5/ppt* mutant embryos (Figures 1K, 1L, 2J, and 2K). A second p53 MO of independent sequence also attenuated the off-target neural death, while a four-base mismatched MO did not show any effect (unpublished data).

### p53 MO as a Tool to Attenuate Neural Cell Death

Because neural death caused by MO-induced off-target effects is so frequent [3,16], we tested the p53 MO as a tool to alleviate off-target neural death. A good tool for this purpose should be effective, innocuous, and specific. The p53 knockdown by itself does not induce any significant defects, as p53 is not required for normal development in mammals or fish [17,18] (Figures 1B and 2B). Also, p53 MO does not affect the efficacy of gene-specific MOs, as it does not interfere with the penetrance of gene-specific phenotypes. To further confirm this, we tested whether p53 MO can affect the efficiency of splicing inhibition by Wnt5 MO1. Semi-quantitative reverse-transcriptase PCR (RT-PCR) analysis of Wnt5 RNA transcripts showed complete blockage of the splicing at exon 5–exon 6 boundary targeted by Wnt5 MO1, which was not affected by p53 MO (Figure 1H).

**Figure 5:**
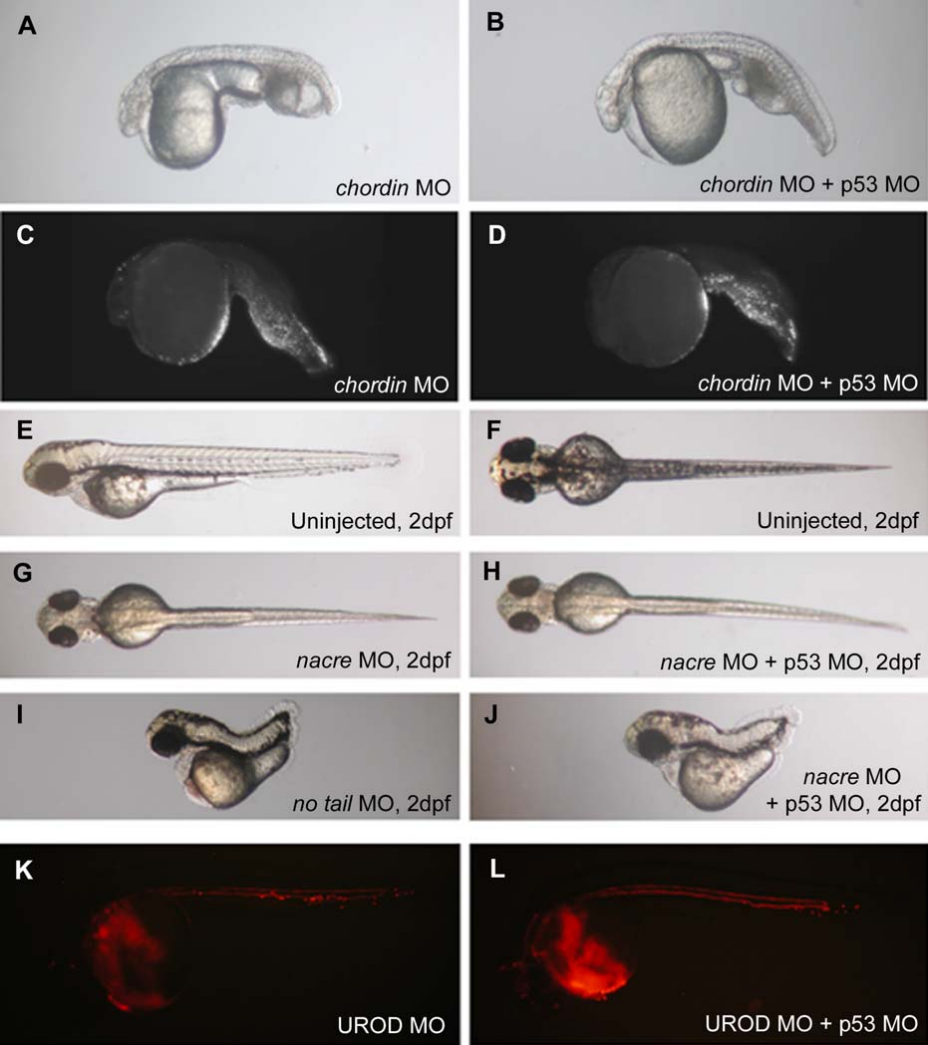
p53 MO Does Not Affect Specific Cell Death or Phenotypes Not Associated with Apoptosis (A–B) Brightfield image of *chordin* morphant (3ng MO) injected (A) or not (B) with p53 MO (2 ng) (1 dpf). (C–D) p53 MO does not affect the localized tail cell death, as also shown by the TUNEL assay (1 dpf). (E–F) Brightfield images of 2 dpf uninjected embryo: lateral (E) and dorsal view (F). (G–H) p53 MO (4 ng) does not affect (H) the lack of dorsal melanophores induced by *nacre* MO (9 ng) (G). (I–J) *no tail* (3 ng) phenotype (I) is not affected by coinjection of 4 ng of p53 MO (J). (K–L) UROD MO (9 ng) induces an autofluorescence of red blood cells (K). This phenotype is not affected by p53 MO coinjection (4 ng) (L) (1 dpf).

We also investigated whether p53 knockdown can affect specific cell death (other than neural death) and whether it affects phenotypes not associated with apoptosis (Figure 5). As shown by morphology and TUNEL staining (Figure 5A–5D), the p53 MO had no effect on the specific tail-cell death induced by the loss of function of *chordin* using a *chordin*-specific MO. In addition, p53 knockdown showed no effect on the MO-induced phenotypes of *nacre* (a pigment defect) (Figure 5G and 5H), *no tail* (a developmental patterning gene) (Figure 5I and 5J), or UROD (loss of function is visualized by fluorescence of red blood cells) (Figure 5K and 5L).

**Figure 6:**
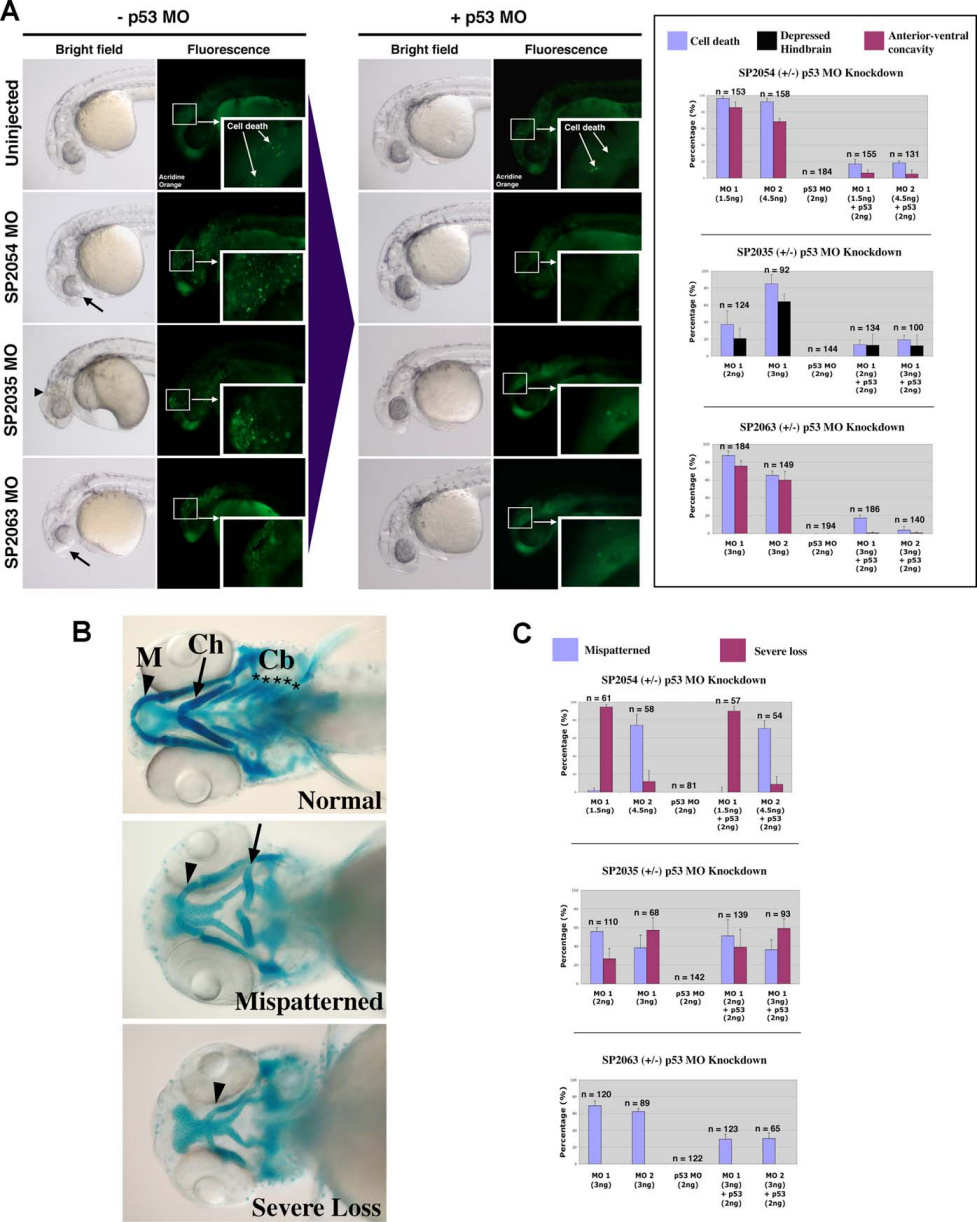
p53 MO Attenuates Cell Death Induced by MOs against Novel Target Genes with Divergent Effects on Craniofacial Phenotypes (A) Brightfield and fluorescent images of 1 dpf embryos injected with MOs targeted against three novel proteins: SP2054, SP2063 (two different MOs for each target: MO1 and MO2), and SP2035. Brightfield images depict observed anterior morphological defects: anterior-ventral concavity (black arrows) and depressed hindbrain (black arrowhead). Fluorescent images indicate areas of apoptosis visualized by live embryo acridine orange staining. Insets show a magnification of apoptotic foci in the head region. Upon p53 coinjection (+p53 MO), there is a significant attenuation of these phenotypes. These results are quantitated for each target in the right graph panel. (B) Brightfield images of 4.5 dpf embryos stained with Alcian Blue to visualize representative craniofacial phenotypes. Arrowheads indicate Meckel's cartilage (M), arrows indicate ceratohyal arch (Ch). Stars indicate the five branchial arches (Cb). Upon MO targeting of the three novel genes, two types of craniofacial phenotypes were observed: mispatterning of the Meckel's cartilage and ceratohyal (mispatterned phenotype), and loss of all branchial arches, ceratohyal and severe hypoplasia of the Meckel's cartilage (severe loss phenotype). (C) Quantitation of the p53 MO effect on craniofacial phenotypes induced by MO targeting of three novel genes. Targeting of SP2054 with MO1 induced a high level of severe loss phenotype, while SP2054 MO2 showed mainly a mispatterned phenotype. None of these craniofacial phenotypes were affected by p53 MO. SP2035 knockdown induced both types of craniofacial abnormalities and the proportion of these was not affected by p53 co-knockdown. SP2063 MOs MO1 and MO2 induced a craniofacial mispatterning phenotype that was partially rescued by p53 MO, suggesting that this craniofacial phenotype is a secondary effect of off-target neural death.

In conclusion, the p53 MO could be an efficient tool to attenuate off-target effects of MOs. We are currently coinjecting the p53 MO with all the MOs tested in a large-scale MO screen [16]. This strategy has greatly attenuated the neural death phenotypes and has notably eased the interpretation of the observed phenotypes, especially in craniofacial development (Figure 6; see below). p53 knockdown or the use of p53 null zebrafish [18] could potentially be of value for use in more traditional genetic approaches, such as chemical or insertional mutagenesis screens, to decrease the collateral tissue damage due to p53-induced cell death that potentially masks important phenotypes of particular interest to investigators.

### p53 MO as a Tool to Facilitate the Study of Craniofacial Development

Early neural death and loss of neural tissue caused by MO off-targeting could potentially affect later craniofacial development. This may generate numerous false positives in MO screening for genes important in craniofacial development. For example, we tested whether p53 co-knockdown could facilitate the analysis of craniofacial phenotypes, especially in the cases of unknown genes or where corresponding mutants are not available. For example, MOs that target three genes in our collection of novel proteins [16], SP2035, SP2054, and SP2063, caused neural death visible at 1 dpf and craniofacial defects visualized by Alcian Blue staining of the cartilage at 4 dpf.

The neural death caused by these MOs was attenuated by p53 co-knockdown (Figure 6A). The brightfield panels in Figure 6A show two types of milder neural defects that we have described in Figure 4; an anterior-ventral concavity for SP2054 and SP2063 (represented by a deficiency in the frontonasal tissue development, black arrows in Figure 6A) and a depressed hindbrain for SP2035 (represented by a lack/developmental delay of the hindbrain tissue, black arrowhead in Figure 6A). Interestingly, these milder defects were clearly associated with neural apoptosis, as shown by acridine orange staining (Figure 6A, fluorescence panels and quantified in corresponding graphs). At higher doses, the MOs against these targets showed a clear cell death pattern even in brightfield images (represented by opaque structures; unpublished data).

Later in development (4 dpf), the MO-injected embryos mentioned above also exhibited craniofacial defects (Figure 6B). We investigated whether these late craniofacial defects were due to the early loss of neural tissue (off-targeting) or to a specific role of the targeted genes in craniofacial development. To achieve this, we analyzed the effect of p53 MO on the cartilage structure at 4 dpf. The craniofacial defects in the SP2035 and SP2054 MO-injected embryos were not affected by p53 co-knockdown, while the craniofacial defects in the SP2063 MO-injected embryo were significantly diminished by p53 co-knockdown (Figure 6C). These results suggest that SP2035 and SP2054 are involved in craniofacial development, while the craniofacial defects seen in SP2063 MO-injected embryo are p53-dependent and thus may be due solely to off-targeting effects of the MO.

**Figure 7:**
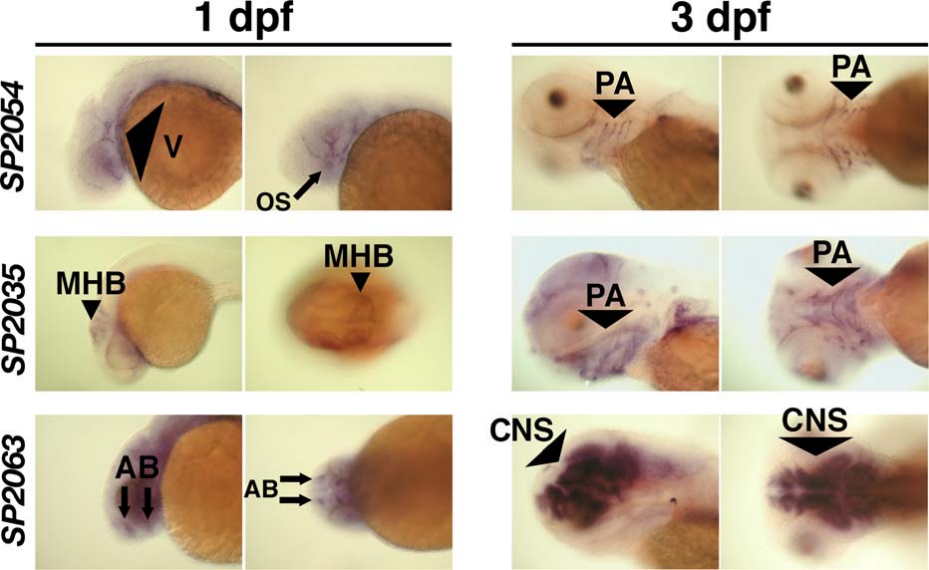
Expression Patterns of SP2035, SP2054, and SP2063 In situ hybridization for SP2054, SP2035, and SP2063 showed that all three transcripts were localized in anterior structures prior to chondrogenesis (1 dpf). Later in development, SP2054 and SP2035 transcripts became localized in pharyngeal arch structures during cartilage formation (2 dpf, see Figure S2; and 3 dpf), while SP2063 mRNA was expressed in brain structures.Please see Figure S2 for a higher resolution version that also includes a 2 dpf time point. AB, anterior brain; CNS, central nervous system; MHB, midbrain/hindbrain boundary; OS, optic stalk; PA, pharyngeal arch; V, vasculature.

To further distinguish putative roles of SP2035, SP2054, and SP2063 in craniofacial development, we analyzed the expression patterns of these genes in zebrafish embryos (Figures 7 and S2). At 1 dpf, all three genes were expressed in the craniofacial region. But while the expression patterns of SP2035 and SP2054 were spatially restricted, SP2063 was more ubiquitously expressed. Interestingly, in subsequent days of development, SP2035 and SP2054 transcripts became specifically enriched in the pharyngeal arches primordia, while SP2063 became restricted to central nervous system structures (Figures 7 and S2). These expression patterns support a direct role of SP2035 and SP2054 in craniofacial development, while the role of SP2063 may be indirect, if any. The CNS expression of SP2063 may also explain the partial rescue of the SP2063 craniofacial phenotypes by p53 MO. If brain structures were affected by SP2063 MO injection, this may have influenced the mechanical structure of cartilage and contributed to the craniofacial phenotype, in conjunction with the loss of neural tissue caused by MO off-targeting.

In conclusion, the likely involvement of the studied novel genes in craniofacial development is supported by their expression pattern and corroborated with the dependence of craniofacial phenotypes on p53. Therefore, p53 co-knockdown can be used to help clarify craniofacial phenotypes induced by MOs against novel genes for which there are no mutant data available for comparison.

### p53 Pathway Is Induced in Morphants with Neural Death

**Figure 8:**
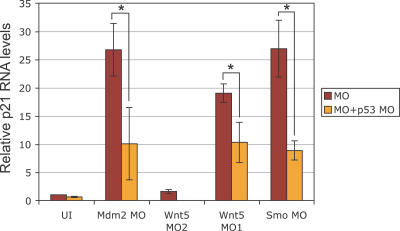
Quantitative RT-PCR of p21/WAF/CIP in Various Morphants p21 RNA levels were significantly increased in MO-injected embryos that showed neural death (Smo MO, Wnt5 MO1, and Mdm2 MO) as compared to the uninjected control (UI). Interestingly, p21 levels were not significantly altered by Wnt5 MO2 (which does not induce neural death). Very importantly, p21 levels were significantly reduced in the respective MO-injected embryos upon p53 co-knockdown (Mdm2 MO *p* < 0.01, Wnt5 MO2 *p* < 0.01, Smo MO *p* < 0.002; *t*-test *p* values for unpaired sets), strongly suggesting that p21 is transcriptionally upregulated by p53 in the morphants with neural death. Error bars indicate standard deviation.

To understand better the mistargeting effects of MOs, we investigated other components of the p53 pathway. A direct target of the p53 transcription factor is p21/WAF/CIP [19]. We tested whether p21 transcription is induced in morphants with neural death. Using quantitative RT-PCR, we observed a significant increase in p21 RNA levels in morphants that show neural death (Smo MO and Wnt5 MO1) but no significant increase in morphants without off-target effects (Wnt5 MO2) (Figure 8). Very importantly, this increase in p21 expression was dependent on p53, since knockdown of p53 significantly decreased p21 RNA levels in respective morphants. Induction of p21 levels provides direct evidence for activation of the p53 protein. These results were similar to the induction of p21 in Mdm2 MO-injected embryos, which was dependent on p53, as expected (Figure 8) [14]. These results suggest that p53 protein is activated by injection of a selection of MOs, associated with off-target neural apoptosis. Consistent with this conclusion, p53 protein is not activated in a selection of morphants that do not exhibit any neural death.

### Transcriptional Regulation of p53 Does Not Contribute Significantly to MO Off-Target Effects

**Figure 9:**
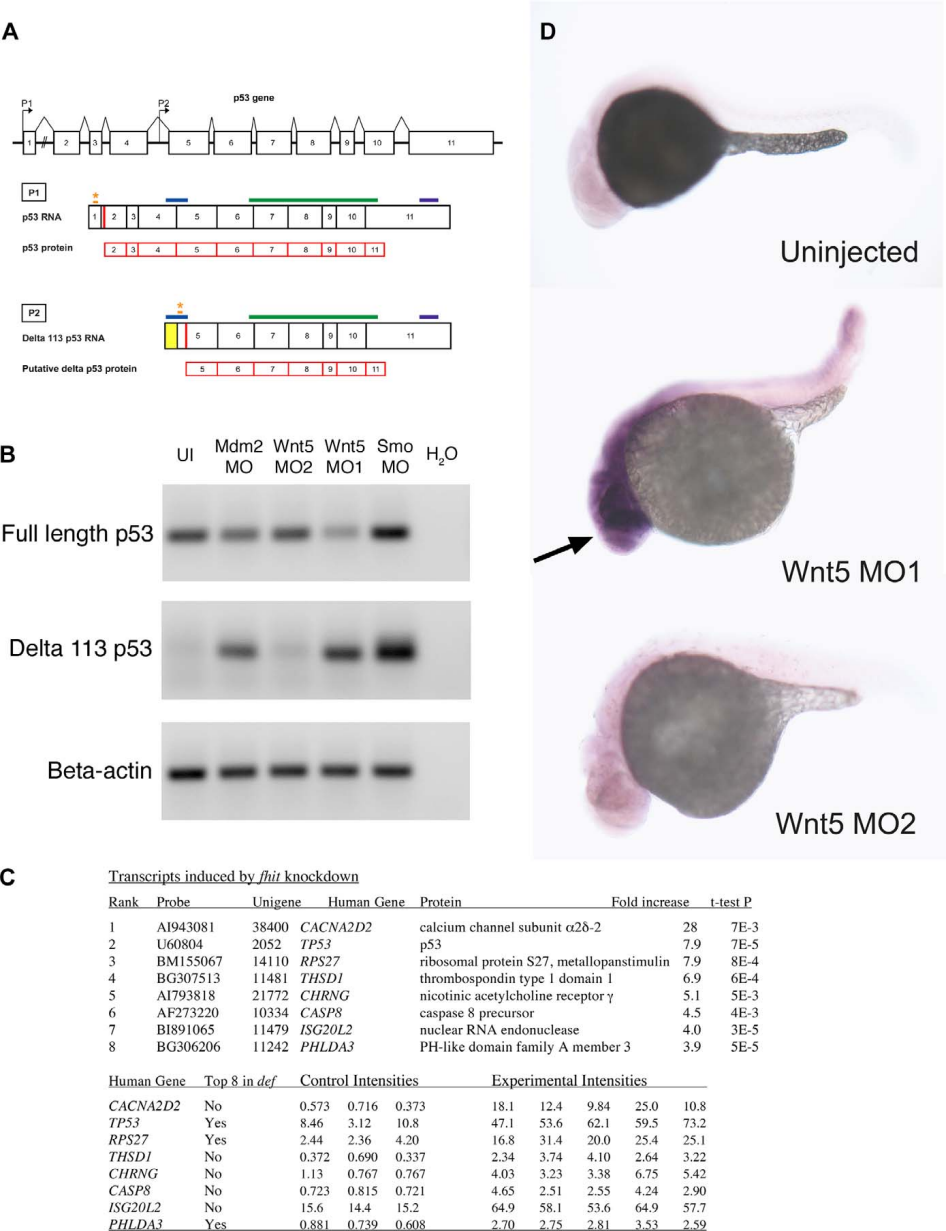
p53 Isoforms Expression in Various MO-Injected Embryos (A) Schematic of p53 and Δ113 p53 transcripts. The p53 gene contains 11 exons (indicated by numbered rectangles), with the two promoters P1 and P2 indicated by the arrows. Transcription initiation at promoter P1 in exon 1 generates the full-length p53 transcript. Δ113 p53 transcript is initiated at an internal promoter in intron 4, P2, and contains the 3′ end of intron 4 (indicated by the yellow rectangle), and likely uses the Met at position 113 as translational start (red bar) [22]. Translation start sites for the two transcripts are indicated by the red bars (in exon 2 for P1; putative translation start in exon 5 for P2, corresponding to Met 113). Translational MO binding sites for the two isoforms are indicated by orange stars. The MO for full-length p53 most likely does not affect the Δ113 p53 transcript, as its binding site is 386 nt upstream of the translational start for the Δ113 p53 transcript. The fragments amplified for the RT-PCR experiments are indicated by the blue bars and are specific to each isoform. The riboprobe fragment for in situ hybridization (indicated by the green bars) and the microarray probe binding site (indicated by the purple bars) are common to both isoforms. (B) RT-PCR for full-length p53 and Δ113 p53. Final products of 30 cycles of RT-PCR for Δ113 p53 were analyzed by gel electrophoresis on a 1% agarose gel, stained with ethidium bromide. β-actin was used as loading control. There is no significant upregulation of full-length p53 expression levels in various morphants. Note the lack of expression of Δ113 p53 in uninjected embryos or morphants with no neural death. Embryos injected with MOs that cause neural death show a significant increase of Δ113 p53 isoform expression. (C) Transcripts induced by *fhit* knockdown in microarray assays. Fold increases in mRNA accumulation and *t*-test *p* values were calculated from the three control (phenol red-injected) intensities and the five experimental intensities determined in eight independent hybridizations using printed 16,399 element 65mer microarrays. The first three experimental samples were embryos injected with the gripNA targeting *fhit* while the fourth and fifth experimental samples were Fhit morphants. NCBI unigene identifiers are for the D. rerio genes. Gene symbols correspond to human orthologs. The three transcripts common to the top eight induced transcripts in *fhit* and *def* [22] datasets are indicated. (D) p53 in situ hybridization. Expression of p53 transcripts is increased in the anterior part of the embryo (indicated by the arrow) upon injection of Wnt5 MO1 (that causes neural death), but not in the morphant with no neural death (Wnt5 MO2).

Translational and post-translational mechanisms of p53 activation have been extensively documented [12]. A well-known mechanism for p53 induction is due to Mdm2 inactivation. Because Mdm2 is a ubiquitin-E3 ligase that targets p53 for proteasomal destruction, loss of function of Mdm2 leads to p53 protein accumulation and consequent apoptosis [14]. We investigated whether p53 transcriptional regulation is part of the p53 induction due to MO off-targeting. The p53 gene is known to express multiple isoforms as result of alternative splicing and internal promoters (Figure 9A) [20]. We designed primers to amplify a fragment specific to full-length p53 cDNA, which is the isoform we targeted by our p53 MO (Figure 9A) and was shown to be sufficient for neural death induction by co-knockdown experiments (Figures 1 and 2). We examined the levels of p53 transcription in embryos injected with various MOs by semi-quantitative RT-PCR. Interestingly, there was no significant increase in full-length p53 RNA levels in various MO-injected embryos (Figure 9B, top panel), suggesting that transcriptional induction of full-length p53 does not play a role in p53 activation by MO off-targeting. These results, together with our observations that knockdown of full-length p53 alleviates MO off-target effects, support a direct role of full-length p53 protein, but not of p53 transcriptional regulation, in neural death caused by MO off-targeting.

### Diagnostic Transcriptional Induction of an N-Terminal Truncated p53 Isoform in Morphants with Off-Target Effects

The p53 locus expresses multiple transcripts as result of alternative splicing and internal promoters [20]. For example, zebrafish have been recently reported to use an internal promoter in intron 4, conserved from flies to humans [21], to express an N-terminal truncated form of p53, Δ113 p53 (Figure 9A) [22]. The truncated p53 isoform is highly upregulated in zebrafish *def* mutants, specifically in the characteristic hypoplastic digestive organs [22].

We tested whether this truncated p53 isoform was induced under cell-death conditions through MO off-targeting. To discriminate between the two p53 transcripts, we designed primers to amplify specifically either the full-length p53 cDNA or that encoding the truncated p53 isoform (Figure 9A) and used semi-quantitative RT-PCR to examine p53 transcripts (Figure 9B). While full-length p53 RNA levels were not significantly increased in any of the MO-injected embryos (Figure 9B, top), the Δ113 p53 isoform was highly upregulated in MO-injected embryos with neural death and virtually absent in the MO-injected embryos with no neural death or in the uninjected controls (Figure 9B, middle).

We also performed microarray screens for the transcriptional consequences of various MOs. As shown in Figure 9C, when zebrafish embryos were treated with a MO or a gripNA [11] against the D. rerio homolog of the *fhit* tumor suppressor gene [23], we obtained evidence for increased transcription at the p53 locus. In five *fhit* knockdown microarrays, p53 transcripts were increased 7.9-fold with respect to control-injected embryos (*t*-test *p*-value = 0.00007). Remarkably, p53 and two other mRNAs among the top eight transcripts induced in the *fhit* datasets were common to the top eight induced genes in *def* zebrafish embryos [22]. Indeed, we have seen many of the same mRNAs coinduced by unrelated MOs (unpublished data). However, it is noteworthy that the probe used for microarrays binds to the 3′ UTR of p53, thus recognizing both full-length and the Δ113 p53 isoforms (Figure 9A).

We also conducted in situ hybridization experiments with a p53 riboprobe in embryos injected with the two Wnt5 MOs, one that showed neural death (MO1) and one that did not (MO2) (Figure 9D). Wnt5 MO1 showed increased p53 mRNA expression in the anterior part of the body (arrow, Figure 9D), while Wnt5 MO2 showed low ubiquitous p53 mRNA expression similar to the uninjected control (Figure 9D). In this case also, the riboprobe could bind both full-length and the Δ113 p53 isoforms (Figure 9A).

The RT-PCR experiments in Figure 9B showed that full-length p53 RNA levels were not increased in any MO-injected embryos, while the Δ113 p53 isoform was highly induced in embryos injected with off-targeting MOs. These results suggest that the increased p53 expression observed by microarray and in situ hybridization consists largely of Δ113 p53 RNA, and that transcriptional induction of full-length p53 does not contribute to p53 activation by MO off-targeting.

### Does Increased Transcription of the Shortened p53 Isoform Cause the MO Off-Targeting Cell Death?

The p53 MO, which blocks neural cell death, was designed to knock down full-length p53 and would not be expected to affect the Δ113 p53 isoform (Figure 9A). To further test whether the highly induced Δ113 p53 mRNA is required for neural cell death, we designed a translational MO to specifically knock down this isoform (Figure 9A). We cannot design a splice-site blocker MO specific only for the N-terminal truncated isoform because all the splice junctions present in Δ113 p53 are also present in full-length p53 (Figure 9A). Coinjection of the Δ113 p53 MO with off-targeting MOs did not block cell death (unpublished data). This result is consistent with the fact that Δ113 p53 lacks the transactivation domain and part of the DNA binding domain, which are thought to be required for induction of apoptosis [21]. Thus, the Δ113 p53 isoform is most likely not the cause of cell death induced by MOs and may represent a diagnostic signature of off-target effects. In contrast, the full-length p53 protein is sufficient to cause neural death due to MO off-target effects, even if transcript levels are unchanged. More experiments are necessary to evaluate the significance of the Δ113 p53 isoform transcriptional induction beyond its use as a diagnostic for p53 activation.

## Discussion

### Mechanism of Off-Target Effects

We have shown that mistargeting MOs induce neural death via a pathway involving p53 activation. Curiously, ongoing synthesis of full-length p53 is required for cell death, while transcription of the Δ113 p53 isoform is a consistent and striking component of the mistargeting MO signature. We investigated various hypotheses for the mechanism underlying this off-target effect. The p53 pathway induction is independent of the intended gene target and appears to be sequence-specific, since two MOs of independent sequence, but targeted to the same gene, have strikingly different effects on p53 induction. This off-target effect is noted in both translational blockers and splice-site MOs, suggesting that the mechanism does not uniquely involve the transcription or the translation machinery. According to our analysis, MOs with off-target effects do not exhibit any overt primary sequence similarity to repeated elements such as rRNA genes or the zebrafish mitochondrial genome (unpublished data).

Although the mechanism of MO-induced p53 activation is still unclear, this pathway is activated by other knockdown technologies including gripNAs (Figures 1I and 1J, 2H and 2I, and 9C). Furthermore, related observations indicate that siRNAs can also induce off-target p53 activation. A recent study reports divergent changes in levels of p53 and p21 in cells subjected to ten different siRNAs targeted to menin [4]. The study shows that, while all the siRNAs knock down menin levels to various extents, some of the siRNAs cause a significant increase in p53 and p21 protein levels, independent of the levels of menin knockdown, while others have no effect on p53 or p21. One hypothesis is that the off-target effects caused by siRNAs are due to short sequence homology to other genes [5–7,24]. We have not observed any pattern of partial homology between off-targeting MOs and p53 or Mdm2 genes (unpublished data).

### Diagnostic Transcriptional Activation of a Shorter Isoform of p53

We have shown that certain MOs and gripNAs induce neural cell death in a manner that depends on synthesis of full-length p53 protein, but not on transcriptional activation of full-length p53. We also observed a diagnostic transcriptional induction of an N-terminal truncated isoform of p53. Interestingly, this truncated form is thought to act as a dominant negative molecule towards full-length p53, as it lacks the transactivation domain and part of the DNA binding domain [21]. The human homolog of Δ113 p53 was shown to be defective in promoting apoptosis and even to inhibit p53-mediated apoptosis [21].

Consistent with these results, a translational MO targeted specifically to the Δ113 p53 isoform did not alleviate the neural death induced by off-targeting MOs (unpublished data), although the full-length p53 knockdown did. Also, overexpression of Δ113 p53 RNA in zebrafish embryos did not cause neural death (unpublished data), suggesting that the Δ113 p53 isoform is insufficient to promote apoptosis. Potentially, the Δ113 isoform is transcriptionally induced secondary to p53-mediated apoptosis.

Transcriptional induction of the Δ113 isoform of p53 may represent a diagnostic signature for a specific type of cellular stress. High levels of the Δ113 isoform p53 transcription were observed in *def* [22] and *fhit* knockdown embryos and in morphants with off-targeting phenotypes, while lower levels of mRNA increase were observed in *flathead* embryos [25]. It remains to be determined whether off-targeting oligos target DNA, an RNA other than mRNA, or another cellular component, and whether the *fhit* knockdown profile is due to off-targeting or to a specific involvement in the stress response pathway.

A previous study also reported the presence of a shorter p53 transcript in zebrafish [14], with a size consistent with the predicted length of the Δ113 p53 isoform. Intriguingly, this shorter transcript was highly upregulated in zebrafish embryos under cell death–inducing conditions such as treatment with camptothecin or roscovitine or knockdown of the anti-apoptotic genes *mdm2* and *tsg1*. Also noteworthy, the expression of the shorter p53 transcript seemed to be dependent on full-length p53 [14].

### Specific versus Off-Target Neural Death

A very important issue for using p53 knockdown to mitigate neural death is specificity. In many cases, neural death can be a specific phenotype, and p53 MO rescue may suggest a specific interaction with the gene of interest. A key experiment to validate a MO phenotype is to observe rescue of the morphant phenotype with an RNA or DNA construct of the respective gene. If the neural death is rescued by the RNA/DNA construct, it is very likely that the gene of interest is specifically involved in cell death. If, however, the RNA/DNA rescue still yields a neural death phenotype, it is possible that the neural death is an off-target effect of the MO. For example, a recent study reported apoptosis and neuronal loss upon knockdown of presenilin enhancer Pen-2 in zebrafish embryos [26]. This neural death was significantly reduced by p53 co-knockdown, as in the case of off-targeting MOs. However, the authors clearly showed a rescue of the neural apoptosis by a Pen-2 RNA construct of a sequence not overlapping with the Pen-2 MO [26]. Together, these results support a true anti-apoptotic role of Pen-2 in promoting neuronal survival.

We have also attempted to rescue the Mdm2 MO-induced cell death phenotype with a Mdm2 RNA construct. However, we did not observe any significant rescue (unpublished data). One potential explanation is that this particular Mdm2 MO also has off-targeting effects. Five additional Mdm2 MOs have been reported to cause cell death that could be rescued by p53 MO [14], but we did not test any of these. We also tested whether the Wnt5 MO1-induced cell death is *wnt5/ppt* specific or a result of off-targeting. There are no indications from previous studies to suggest a role of *wnt5/ppt* in cell death. We observed no effect of a *wnt5/ppt* RNA construct [27] on the cell death specifically induced by Wnt5 MO1 (unpublished data), but not by Wnt5 MO2. However, we did not observe any rescue of the characteristic morphological defect associated with loss of *wnt5/ppt* either. This result is not so surprising, though, as there is no previous report on a successful RNA rescue of the body axis shortening phenotype caused by *wnt5/ppt* inactivation (either mutation- or MO-induced). It is possible that generalized overexpression of *wnt5/ppt* RNA may not be sufficient to compensate for decreased *wnt5/ppt* levels at the appropriate time and place.

In conclusion, if a cell-death phenotype caused by knockdown can be rescued by the respective RNA/DNA construct, it is likely that the gene of interest is involved in cell survival. If the RNA/DNA construct rescues the gene-specific phenotype but does not rescue the cell death phenotype observed in MO-injected embryos, it is likely that cell death represents an off-targeting effect of the MO. It is also possible that certain MO-induced phenotypes cannot be rescued by corresponding RNA/DNA overexpression, due to improper localization and/or timing of expression during development.

Ongoing work is geared to exploit p53 co-knockdown to alleviate off-target neural death of MOs and to discover the mechanism by which off-target MOs induce p53 activation as well as the signature Δ113 p53 transcript. Potentially, p53 knockdown by RNAi may also alleviate the off-target effects of siRNAs [7].

## Materials and Methods

### Fish maintenance.

Wild-type zebrafish were purchased from Segrest farms (http://www.segrestfarms.com). Embryos were raised at 30 °C and spawning was carried out as described [28]. *wnt5* mutant fish carrying the *ppt* ^hi1789b^ allele [29] were obtained from the Zebrafish International Resource Center (http://zebrafish.org/zirc).

### MO and gripNA sequences and injections.

MO and gripNA sequences are shown in Table 1. MOs were obtained from Gene Tools (http://www.gene-tools.com) and were prepared and injected in 1–4 cell stage embryos as described [30]. When two MOs were injected in the same embryo, we carried out both separate injections of the different MOs and single injections of MO mixtures, with very similar results. The only difference was a slightly increased mortality in the case of double-injected embryos as compared to single injections. In all cases, except where noted, p53 MO was injected 1.5-fold (w/w) to the other MO used. MO doses were: 3 ng of Smo MO, Wnt5 MO1, and Mdm2 MO; 4.5ng of p53 MO (except where noted otherwise); and 6ng of Wnt5 MO2. GripNAs were obtained from Active Motif (http://www.activemotif.com) and were prepared and injected similar to MOs. Wnt5 GripNA was injected at 2.25 ng, and coinjections with p53 MO were at 4 ng.

**Table 1:**
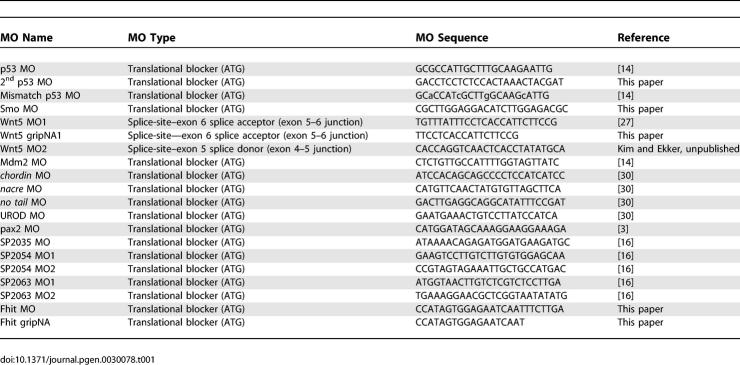
MO Sequences

Embryos were visualized at 24–29 hpf, except where noted. Microscopy was performed on a Zeiss Axioplan 2 microscope (http://www.zeiss.com) fitted with differential interference contrast microscopy optics. Images were captured with a Nikon Coolpix 995 (http://www.nikonusa.com) or a Canon PowerShot G6 digital camera (http://www.canon.com), with multiple images combined using Adobe Photoshop software (http://www.adobe.com).

### TUNEL assay.

Embryos were dechorionated and fixed at 30 hpf or as indicated in 4% paraformaldehyde for 1 h at room temperature. They were then washed with PBS buffer twice and permeabilized with 0.1% sodium citrate and 0.1% TritonX for 2 min on ice. After washing twice in PBS buffer, embryos were incubated with the reaction mixture containing the terminal deoxynucleotidyl transferase and TMR-labeled nucleotides for 1 h in the dark at 37 °C. Reaction was stopped by washing with PBS three times. Terminal deoxynucleotidyl transferase catalyzes incorporation of labeled nucleotides to 3′-OH DNA ends in a template-independent reaction. The fluorescent signal was visualized and imaged using a Zeiss Axioplan 2 microscope coupled to an ApoTome, using AxioVision 4.2 software. z-stacks were superimposed using Extended Focus feature of the software.

### Acridine orange assay.

Live embryos were immersed in 5 μg/ml acridine orange (Sigma, http://www.sigmaaldrich.com) for 10 min, then visualized and imaged for less than 60 s (the signal is quenched after 60-s exposure to fluorescence), as described for the TUNEL assay.

### Quantitative RT-PCR.

Total RNA was extracted from 32 hpf embryos using TRIZOL reagent (Invitrogen, http://www.invitrogen.com). Quantitative RT-PCR was carried out on 200ng of RNA using the LightCycler RNA Amplification kit SYBR Green (Roche, http://www.roche-diagnostics.us) in a LightCycler 2.0 Instrument, following manufacturer's protocols. The primers used are shown in Table 2. All expected PCR products span at least one intron (except the Δ113 p53 fragment), to ensure amplification solely from the cDNA and not from the genomic DNA. The primers for full-length p53 correspond to exon 4 (not present in the Δ113 p53 isoform) and exon 5. The primers for the Δ113 p53 isoform correspond to intron 4 (not present in the full length p53) and exon 5. The identity of the RT-PCR products was confirmed by sequencing. The samples were quantified by comparative cycle threshold (Ct) method for relative quantification of gene expression [31], normalized to β-actin. All experiments were performed with at least two different RNA preparations and at least three independent experiments for each RNA preparation.

**Table 2:**
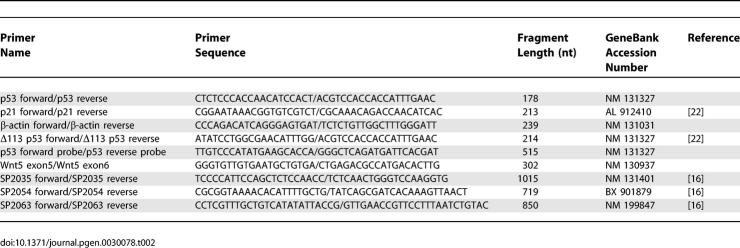
Primer Sequences

### In situ hybridization.

cDNA for p53 probe was amplified using total RNA from 24 hpf zebrafish embryos injected with pax2 MO (Table 1) with primers shown in Table 2. The p53 riboprobe used in the in situ hybridization experiments spans exons 6–11, a region common to both full-length and Δ113 p53 isoforms. The cDNAs for SP2035, SP2054, and SP2063 were amplified from total RNA from 30 hpf zebrafish embryos, using primers indicated in Table 2. The PCR fragments for p53, SP2035, SP2054, and SP2063 were cloned into the pCRII TOPO vector (Invitrogen). The plasmids were then linearized with NotI (p53, SP2054 and SP2063) or Spe I (SP2035). DIG-labeled antisense RNA was synthesized using the SP6 polymerase (p53, SP2054, and SP2063) or T7 polymerase (SP2035) in conjunction with the in vitro DIG labeling kit (Roche). Zebrafish in situ hybridization was performed on 26–28 hpf embryos or indicated time points as previously described [32]. Microscopy was performed on a Zeiss Axioplan 2 microscope using DIC optics. Images were captured with a Canon PowerShot G6 digital camera.

### Alcian Blue cartilage staining.

Cartilage was stained with Alcian Blue using a modification of previously published protocols [33,34]. Anesthetized 4.5 dpf larvae were fixed in 4% phosphate-buffered paraformaldehyde overnight at 4 °C, then stained with 0.1% Alcian Blue (Sigma) in 70% ethanol and 0.37% hydrochloric acid for 4–6 hours at 4 °C. The embryos were cleared in 70% ethanol and 0.37% hydrochloric acid mixture, then rehydrated stepwise in PBS buffer. To enhance optical clarity, embryos were bleached with 3% H_2_O_2_ and 1% KOH for 20 min, then washed with PBS containing 0.2% Tween-20, then with PBS, and lastly with H_2_O. Embryos were stored in 50% glycerol with 0.25% KOH at 4 °C and were mounted in 2% methylcellulose for imaging.

### Microarrays.

Transcriptional profiling was performed by the Thomas Jefferson University Microarray Facility at the Kimmel Cancer Center. The spotted array contains 16,399 oligonucleotides (Compugen; annotated at http://giscompute.gis.a-star.edu.sg/~govind/zebrafish/version2). More than 100 β-actin oligonucleotides that serve as positive controls were present on each chip.

Zebrafish embryos were injected with phenol red control or 0.5 nl of 1 mM Fhit MO or 1 nl of 1 mM Fhit gripNA. Total RNA of 24 hpf phenol red control and MO-injected embryos were extracted by TRIZOL (Invitrogen). Gene expression was determined using biotin-labeled and in vitro–transcribed antisense RNA generated from the total RNA template. Each chip was scanned and quantified using a ScanArray Express laser scanner (PerkinElmer, http://www.perkinelmer.com). The signals on the oligo microarray were normalized by the median and regularized *t*-test was performed to determine significant differences between the controls and morphants. The p53 probe used in the microarrays corresponds to a short EST in the 5′ UTR of the gene (U60804) and consequently is common to both full-length and the Δ113 p53 isoform.

## Supporting Information

Figure S1 Temporal and Spatial Characterization of Representative MO-Induced Neural Cell Death during Early Embryogenesis (A) Brightfield and darkfield images of Wnt5 MO1-injected embryos. 14 hpf (A–I), 22 hpf (J–R), and 30 hpf (S–AA). Uninjected embryos (A–C, J–L, and S–U), intermediate cell death phenotype (D–F, M–O, and V–X), and severe cell death phenotype (G–I, P–R, and Y–AA). Lateral views (A, D, G, J, M, P, S, V, and Y), all others dorsal head views. Intermediate cell death is observed at 14 hpf as highly localized opaque cells in the head (large arrow in D), which are arranged near the lateral (arrowhead in E and F) and midline (small arrow in E and F) areas of the developing brain. This pattern progresses through 22 and 30 hpf (M–O and V–X), including a concentration of opaque cells surrounding the emerging folds of the brain midline (small arrows N–O and W–X) and the eye (arrowheads N–O and W–X). Severe cell death is observed as highly dense areas of opaque cells throughout the developing head.
(B) TUNEL assay. Zebrafish embryos were injected with Wnt5 MO1 and analyzed by TUNEL assay at 14 hpf (A–F), 22 hpf (G–O), and 30hpf (P–X) stages. Uninjected embryos: A–C, G–I, and P–R. At the later time points two classes of phenotypes were observed: an intermediate (J–L and S–U) and a severely affected class of embryos (M–O and V–X). These were characterized by intense fluorescent apoptotic foci in the head and body, with increasing intensity corresponding to increased severity (higher MO dose). This figure represents a higher resolution version of Figure 3.
(5.4 MB TIF)

Figure S2 Expression Patterns of SP2035, SP2054, and SP2063 In situ hybridization for SP2054, SP2035, and SP2063 showed that all three transcripts were localized in anterior structures prior to chondrogenesis (1 dpf). Later in development, SP2054 and SP2035 transcripts became localized in pharyngeal arch structures during cartilage formation (2 dpf and 3 dpf), while SP2063 mRNA was expressed in brain structures.
AB, anterior brain; CNS, central nervous system; MHB, midbrain/hindbrain boundary; OS, optic stalk; PA, pharyngeal arch; V = vasculature.
(9.5 MB TIF)

### Accession Numbers

Accession numbers for the genes and gene products from the Ensembl D. rerio genome database (http://www.ensembl.org/Danio_rerio/index.html) are β-actin, NM 131031; *chordin,* NM 130973; Mdm2, NM 131364; *nacre,* NM 130923; *no tail,* NM 131162; p21, AL 912410; p53, NM 131327; pax2, NM 131184; *smoothened,* NM 131027; SP2035, NM 131401; SP2054, BX 901879; SP2063, NM 199847; UROD, NM 131347; and *wnt5,* NM 130937.

## Abbreviations

dpf: days post-fertilization
hpf: hours post-fertilization
MO: Morpholino phosphorodiamidate oligonucleotide
RT-PCR: reverse-transcriptase PCR
siRNA: small inhibitory RNA
TP53: p53 tumor suppressor gene
TUNEL: transferase-mediated dUTP nick end labeling
