# Supplementary figures and images for: p53 Activation by Knockdown Technologies
Source: PLoS Genet. 2007 May 25;3(5):e78. doi: 10.1371/journal.pgen.0030078 (PMC1877875; doi:10.1371/journal.pgen.0030078)

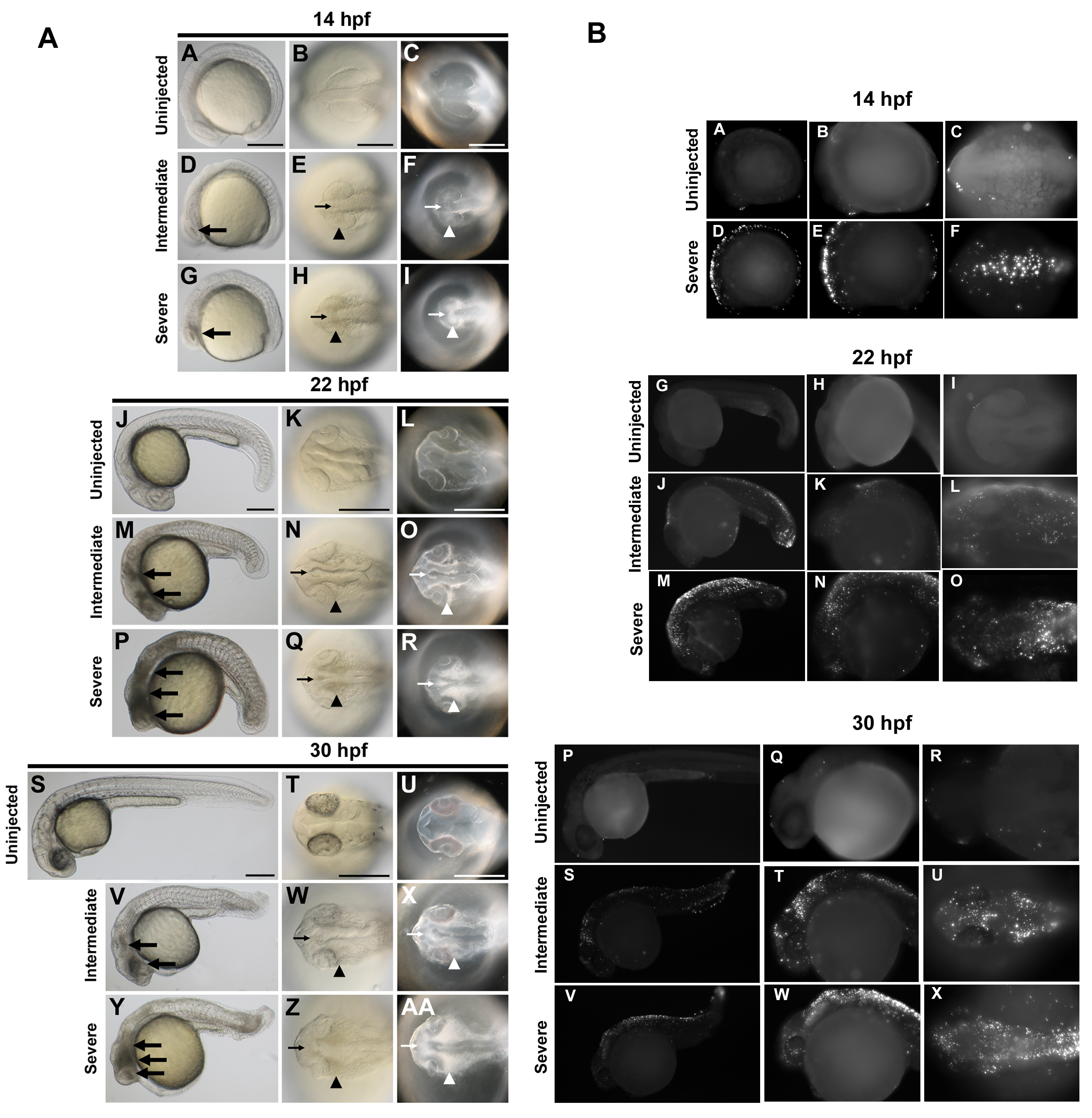

Supplement: Figure S1 — (A) Brightfield and darkfield images of Wnt5 MO1-injected embryos. 14 hpf (A–I), 22 hpf (J–R), and 30 hpf (S–AA). Uninjected embryos (A–C, J–L, and S–U), intermediate cell death phenotype (D–F, M–O, and V–X), and severe cell death phenotype (G–I, P–R, and Y–AA). Lateral views (A, D, G, J, M, P, S, V, and Y), all others dorsal head views. Intermediate cell death is observed at 14 hpf as highly localized opaque cells in the head (large arrow in D), which are arranged near the lateral (arrowhead in E and F) and midline (small arrow in E and F) areas of the developing brain. This pattern progresses through 22 and 30 hpf (M–O and V–X), including a concentration of opaque cells surrounding the emerging folds of the brain midline (small arrows N–O and W–X) and the eye (arrowheads N–O and W–X). Severe cell death is observed as highly dense areas of opaque cells throughout the developing head. (B) TUNEL assay. Zebrafish embryos were injected with Wnt5 MO1 and analyzed by TUNEL assay at 14 hpf (A–F), 22 hpf (G–O), and 30hpf (P–X) stages. Uninjected embryos: A–C, G–I, and P–R. At the later time points two classes of phenotypes were observed: an intermediate (J–L and S–U) and a severely affected class of embryos (M–O and V–X). These were characterized by intense fluorescent apoptotic foci in the head and body, with increasing intensity corresponding to increased severity (higher MO dose). This figure represents a higher resolution version of Figure 3. (5.4 MB TIF) [file pgen.0030078.sg001.tif]

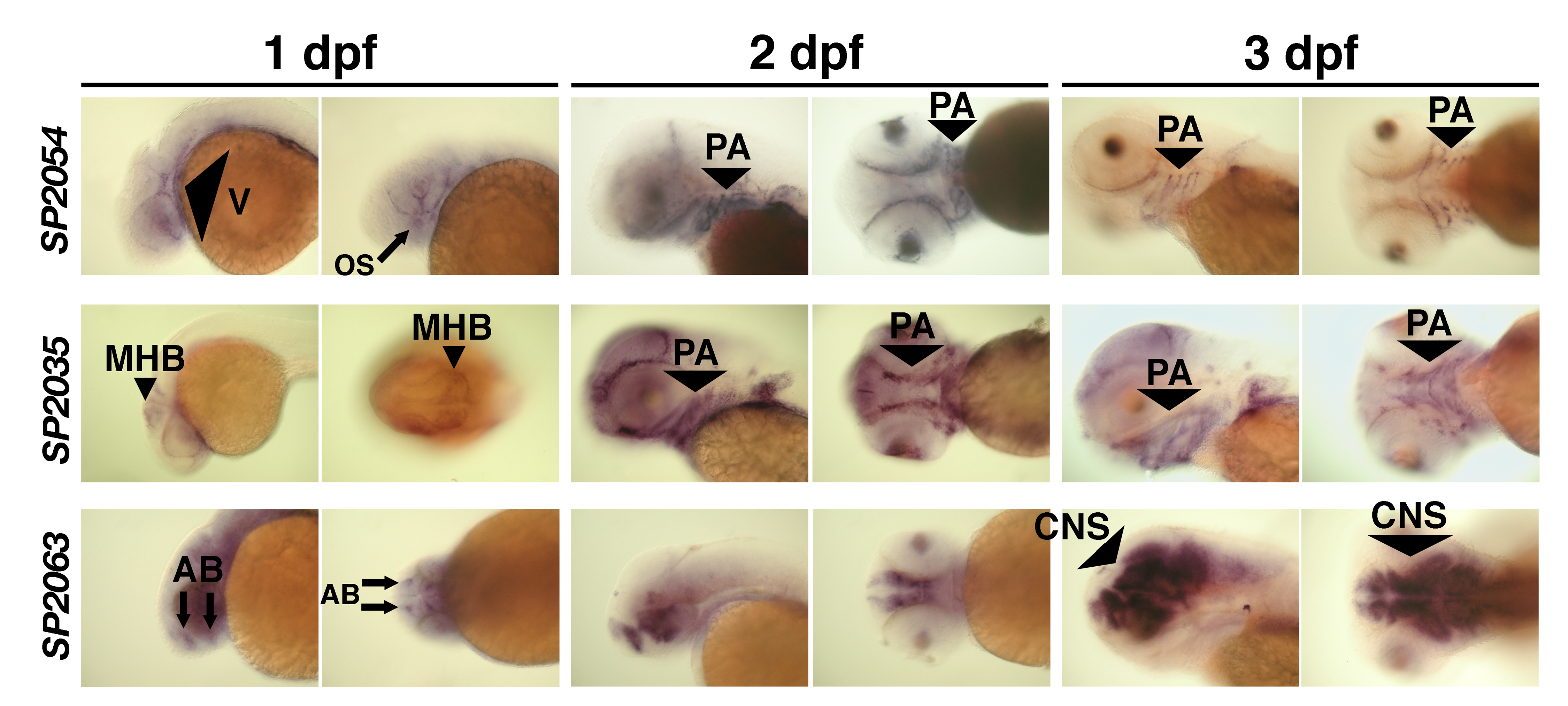

Supplement: Figure S2 — In situ hybridization for SP2054, SP2035, and SP2063 showed that all three transcripts were localized in anterior structures prior to chondrogenesis (1 dpf). Later in development, SP2054 and SP2035 transcripts became localized in pharyngeal arch structures during cartilage formation (2 dpf and 3 dpf), while SP2063 mRNA was expressed in brain structures. AB, anterior brain; CNS, central nervous system; MHB, midbrain/hindbrain boundary; OS, optic stalk; PA, pharyngeal arch; V = vasculature. (9.5 MB TIF) [file pgen.0030078.sg002.tif]
